# Cerebellar Intermittent Theta-Burst Stimulation Reduces Upper Limb Spasticity After Subacute Stroke: A Randomized Controlled Trial

**DOI:** 10.3389/fncir.2021.655502

**Published:** 2021-10-27

**Authors:** Yi Chen, Qing-Chuan Wei, Ming-Zhi Zhang, Yun-Juan Xie, Ling-Yi Liao, Hui-Xin Tan, Qi-Fan Guo, Qiang Gao

**Affiliations:** ^1^Department of Rehabilitation Medicine, West China Hospital, Sichuan University, Chengdu, China; ^2^Key Laboratory of Rehabilitation Medicine in Sichuan Province, Chengdu, China; ^3^Department of Ultrasound Medicine, West China Hospital, Sichuan University, Chengdu, China; ^4^Daping Hospital, Third Military Medical University, Chongqing, China

**Keywords:** stroke, spasticity, intermittent theta-burst stimulation, upper limb, randomized controlled trial

## Abstract

**Objective:** This study aims to explore the efficacy of cerebellar intermittent theta-burst stimulation (iTBS) on upper limb spasticity in subacute stroke patients.

**Methods:** A total of 32 patients with upper limb spasticity were enrolled and randomly assigned to treatment with cerebellar iTBS or sham stimulation before conventional physical therapy daily for 2 weeks. The primary outcomes included the modified Ashworth scale (MAS), the modified Tardieu scale (MTS), and the shear wave velocity (SWV). The secondary outcomes were the *H*-maximum wave/*M*-maximum wave amplitude ratio (*H*_max_/*M*_max_ ratio), motor-evoked potential (MEP) latency and amplitude, central motor conduction time (CMCT), and the Barthel Index (BI). All outcomes were evaluated at baseline and after 10 sessions of intervention.

**Results:** After the intervention, both groups showed significant improvements in the MAS, MTS, SWV, and BI. In addition, patients treated with cerebellar iTBS had a significant increase in MEP amplitude, and patients treated with sham stimulation had a significant decrease in *H*_max_/*M*_max_ ratio. Compared with the sham stimulation group, the MAS, MTS, and SWV decreased more in the cerebellar iTBS group.

**Conclusion:** Cerebellar iTBS is a promising adjuvant tool to reinforce the therapeutic effect of conventional physical therapy in upper limb spasticity management after subacute stroke (Chinese Clinical Trial Registry: ChiCTR1900026516).

## Introduction

Poststroke spasticity (PSS) is a motor disorder clinically manifested as a velocity-dependent increase in stretch reflexes due to the hyperexcitability of alpha motor neurons in the spinal cord (Ward, 2012). It is one of the most common complications after stroke, affecting 19–43% of survivors (Aloraini et al., 2015; Cai et al., 2017). Around 36% of the patients suffered moderate or severe upper limb spasticity (Nam et al., 2019). Weakness, pain, loss of dexterity, stiffness, fibrosis, and atrophy followed by upper limb PSS always contribute to disordered motor control, functional limitations, and poor quality of life that result in an increased burden on caregivers (Leo et al., 2017; Li et al., 2019).

The treatments for managing PSS include pharmacological and non-pharmacological options (Bethoux, 2015). Oral medications are well-known and generally safe, but usually with side effects, especially sedation and weakness (Dvorak et al., 2011). Botulinum toxin A injection, the most widely used local management of spasticity, has a definite efficacy on severe PSS but has a more limited impact on function (Kinnear et al., 2014; Bethoux, 2015). Conventional physical therapy, one of the non-pharmacological options, is strongly recommended for patients with clinically significant spasticity (Khan et al., 2019).

Repetitive transcranial magnetic stimulation (rTMS) is one of the physical therapies for spasticity management, and the efficacy of rTMS on upper limb spasticity after stroke has been identified over the past 10 years (Barros Galvao et al., 2014; McIntyre et al., 2018). Intermittent theta-burst stimulation (iTBS) is a novel form of rTMS, which was developed by John Rothwell in his laboratory in 2005 (Rounis and Huang, 2020). It can lead to consistent and long-lasting therapeutic effects in regulating the excitability of neural structures (Huang et al., 2005). Previous studies have confirmed that iTBS can decrease upper limb spasticity and improve motor function in individuals with stroke (Kim et al., 2015; Chen et al., 2019).

The cerebellum works in concert with the cerebral cortex and plays an important role in muscle tone adjustment (Glickstein et al., 2009). The corticopontocerebellar pathway and the cerebellothalamocortical system are the two main connections. In addition, the cerebellum as a promising stimulation target of neuromodulation has been investigated by accruing studies in recent years. Relevant research revealed that the cerebellum suppresses cortical excitability of the motor cortex via cerebellar brain inhibition (CBI) (Fernandez et al., 2018). Koch et al. found that cerebellar iTBS has efficacy in reconstructing cerebello-cortical plasticity and recovering motor function in individuals with stroke (Koch et al., 2018). However, no study has been conducted to investigate the effect of cerebellar iTBS on PSS since now. Therefore, the objective of this study was to preliminarily explore the short-term efficacy of cerebellar iTBS coupled with conventional physical therapy on upper limb spasticity in subacute stroke patients. We hypothesized that iTBS over ipsilesional cerebellum combined with conventional physical therapy could improve PSS significantly more than by applying conventional physical therapy alone.

## Materials and Methods

### Participants

A total of 32 patients [25 males (78%); mean (SD) age, 54.14 (9.02) years] were enrolled from Sichuan University West China Hospital Rehabilitation Medicine (Chengdu, Sichuan Province, China) between September 2019 and September 2020. Inclusion criteria were as follows: (1) age between 18 and 80 years (Koch et al., 2018); (2) first-ever unilateral ischemic or hemorrhagic stroke confirmed by computed tomography or magnetic resonance imaging; (3) subacute stroke survivors (stroke onset ranged from 2 weeks to 6 months) (Chien et al., 2020; Soulard et al., 2020); (4) having affected elbow flexors and wrist flexors spasticity with the modified Ashworth scale (MAS) score between 1+ and 3 (Barros Galvao et al., 2014); and (5) absence of cognitive impairment that is determined by the mini-mental state examination score is over 27 (Sun et al., 2014). Exclusion criteria included the following: (1) coexisting other neurological diseases; (2) cerebellar or brain stem injury; (3) used anti-spasticity drugs or injected botulinum toxin type A within 3 months before enrollment; (4) severe general impairment or concomitant diseases; and (5) contraindications for rTMS (e.g., history of seizures, intracranial metallic implants, cardiac pacemakers, and pregnancy).

### Trial Design

This randomized, double-blind, sham-controlled trial was designed to explore the safety and the short-term efficacy of cerebellar iTBS on upper limb spasticity after subacute stroke. Eligible participants were randomly allocated in a 1:1 ratio to either cerebellar iTBS group or sham stimulation group. The randomization sequences were generated based on the table of random digits and were concealed in opaque numbered envelopes, which were opened in numerical order by a neutral non-involved researcher. All outcome measures were evaluated at baseline (T0) and after 10 sessions of intervention (T1). Each evaluation was performed by a clinician or by a physical therapist who was blinded to the experimental condition of the patient. Patients themselves were also unaware of the group assignment.

The sample size was estimated using G^*^power of 3.1.9.2 (Faul et al., 2007), with the following parameters: effect size (*d*) = 1.35, α = 0.05 (two tails), power (1–β) = 90%, and allocation ratio *n*2/*n*1 = 1. The effect size was determined based on the result of our pilot study. After calculation, the necessary sample size of *n* = 26 was obtained. Considering the compliance of subjects, a total of 32 patients were required to allow for a 20% dropout rate.

### Ethics Committee

This study was approved by the local institutional biomedical Ethics Committee on September 30, 2019, and complied with the Declaration of Helsinki. All patients provided written informed consent before the experiment. The trial was then registered on October 13, 2019, in the Chinese Clinical Trial Registry (registration number: ChiCTR1900026516).

### Interventions

Each patient received 1 session of cerebellar iTBS or sham stimulation daily, always before conventional physical therapy, for a total of 10 sessions. The overall intervention periods were 5 days/week for 2 consecutive weeks. All the patients did not use anti-spasticity drugs or injected botulinum toxin type A throughout the whole trial.

#### Intermittent Theta-Burst Stimulation

Before each daily conventional physical therapy, one session of cerebellar iTBS was applied over the ipsilesional lateral cerebellum, which was carried out using a 70-mm figure 8 coil attached to a magnetic stimulator (Yiruide Medical Company, Wuhan, China). The coil was positioned tangentially to the scalp, with the handle pointing superiorly. The center of the coil was positioned at 1 cm inferior and 3 cm lateral to the inion based on previously reported studies (Hardwick et al., 2014; Olfati et al., 2020). The stimulation intensity was determined by the active motor threshold (aMT), defined as the lowest intensity which evoked at least 5 out of 10 motor-evoked potentials (MEP) with an amplitude >200 μV peak to peak in the abductor pollicis brevis muscle during 10% of maximum contraction (Popa et al., 2013; Koch et al., 2019). iTBS protocol was used with a total of 600 pulses over 200 s delivered at 80% aMT (Schwippel et al., 2019). For sham stimulation, the stimulation coil was rotated 90° so that the minimal current flow was induced in the brain, and it was still centered on the same scalp position with the same parameter as the cerebellar iTBS group (Wang et al., 2020).

#### Conventional Physical Therapy

Conventional physical therapy program was composed of exercises designed to improve spasticity and promote recovery of voluntary motor function of the upper limb, including limb positioning, postural training, stretching, task-oriented therapy, and sensory stimulation (Winstein et al., 2016; Kucukdeveci et al., 2018), lasting 50 min per session (Supplementary Material).

### Outcome Measures

#### Primary Outcome Measures

The primary outcomes were the measurements to assess the elbow flexors and wrist flexors spasticity of the affected upper limb, including the modified Ashworth scale (MAS), the modified Tardieu scale (MTS), and the shear wave velocity (SWV).

##### Modified Ashworth Scale

The MAS is a reliable scale for evaluating the muscle tone in individuals with stroke, which has shown satisfactory inter- and intra-rater reliability and agreement (Meseguer-Henarejos et al., 2018; Chen et al., 2019). It was scored using a six-point (0, 1, 1+, 2, 3, 4) scale, ranging from 0 (normal muscle tone) to 4 (limb rigid in flexion or extension) (Li et al., 2020).

##### Modified Tardieu Scale

The MTS measures spasticity using three parameters: angle of fast-stretch R1, angle of relatively slow-stretch R2, and angle differences between R2 and R1 (Ben-Shabat et al., 2013). The differences between R2 and R1 indicate the level of the dynamic component of spasticity in the muscle (Singh et al., 2011). A standard goniometer was utilized to measure the range of motion of the elbow and wrist joints.

##### Shear Wave Ultrasound Elastography

The muscle hardness of the affected biceps brachii and flexor carpi radialis was measured at a relaxed position using the shear wave ultrasound elastography images obtained by an ultrasonic apparatus (Resona 7, Mindray, Shenzhen, China). The transducer (L9-3U type) was placed over the bellies of biceps brachii or flexor carpi radialis and was perpendicular to the muscle fibers in the transverse axis (Wu et al., 2017). In the image, a region of interest (ROI, 0.5 cm^*^0.5 cm) was set near the center part where the muscle was thickest. During SWV acquisition, a warm thin layer of acoustic gel was kept on the skin and the transducer was held stationary. After that, three measurements of SWV with the lowest coefficient of variation were acquired and averaged for further statistical analysis (Akagi and Takahashi, 2013).

#### Secondary Outcome Measures

The secondary outcomes included the H-maximum wave/M-maximum wave amplitude ratio (*H*_max_/*M*_max_ ratio) to assess the intrinsic excitability of the alpha motor neurons, neurophysiological parameters to assess the cortical activity, and the Barthel Index (BI) to assess the ability of activities of daily living (ADL).

##### H_***max***_/M_***max***_ Ratio

Compound muscle action potentials and Hoffmann (*H*) reflex were obtained using an electromyography (EMG) unit (Keypoint, Dantec, Denmark) with a bandpass filter at 20 Hz to 2 kHz, sweep speed at 10 ms/division, and sensitivity at 200–500 μV. Ag–AgCl surface electrodes were utilized to record the EMG activity. A bipolar stimulus probe was used to stimulate the median nerve at the antecubital fossa. After skin preparation, the active electrode was placed over the bellies of the affected flexor carpi radialis at one-third of the proximal distance between the medial epicondyle of the humerus and the radial styloid, the reference electrode was 4 cm distal and lateral to the active electrodes, and the ground electrode was between the stimulating and the active electrode (Pizzi et al., 2005; Naghdi et al., 2014). Stimulus intensity was gradually increased until an H-reflex emerged. And then, the *H*_max_/*M*_max_ ratio was recorded to estimate the intrinsic excitability of the alpha motor neurons.

##### Neurophysiological Parameters

Neurophysiological parameters were recorded by the above-mentioned TMS instrument. The MEP latency and amplitude, and central motor conduction time (CMCT) were detected in the unaffected hemisphere recorded by the contralateral abductor pollicis brevis. Before the MEP measurement, the resting motor threshold (rMT) was determined as the lowest intensity to evoke at least 5 MEPs of peak-to-peak amplitude higher than 50 μV on 10 consecutive stimulations during a resting period. Later, latencies and amplitudes of 5 MEPs were obtained by stimulating at 120% of the rMT intensity (Pisa et al., 2020), and three intermediate values were acquired and averaged for further statistical analysis. In addition, the CMCT was calculated as the latency difference between MEPs elicited by stimulating the motor cortex and those evoked by spinal (motor root) stimulation (Cakar et al., 2016).

##### Barthel Index

The BI is a 10-item measure of ADL (i.e., feeding, bathing, personal hygiene, dressing, bowel control, bladder control, using the toilet, chair/bed transfer, ambulation, and stair climbing) used to quantify functional change after rehabilitation intervention (Silveira et al., 2018). It is a self-reported scale with excellent inter-rater reliability for standard administration after stroke (Duffy et al., 2013).

### Statistical Analysis

Professional physical therapists monitored adverse effects throughout the trial. Data management and analyses were performed using GraphPad Prism 7.00 (GraphPad Software, Inc., La Jolla, CA, USA). Before undergoing statistical analyses, the normal distribution of data was evaluated by the Shapiro–Wilk test. Continuous variables, ordinal variables, and categorical variables were, respectively, presented as mean (±standard deviation, SD), medians (interquartile range, IQR), and number (percentage, %). The level of significance was set at α = 0.05. Descriptive analyses were conducted to show the demographic and clinical characteristics of subjects. Fisher's exact test and unpaired *t*-test were used to evaluate the differences between the groups in the distribution of the characteristics of the subject at baseline.

For the MAS, the scores 0, 1, 1+, 2, 3, and 4 were converted to 0, 1, 2, 3, 4, and 5. The intervention efficacy within the group and between groups was analyzed with the Wilcoxon matched-pairs signed-rank test and the Mann–Whitney *U* test, respectively. For other outcome measures, a paired *t*-test of raw data was performed to evaluate the treatment effects within groups. Additionally, an unpaired *t*-test of changes between T0 and T1 was conducted to analyze the difference between groups.

## Results

### Participant Characteristics and Flow of the Trial

**Figure 1:**
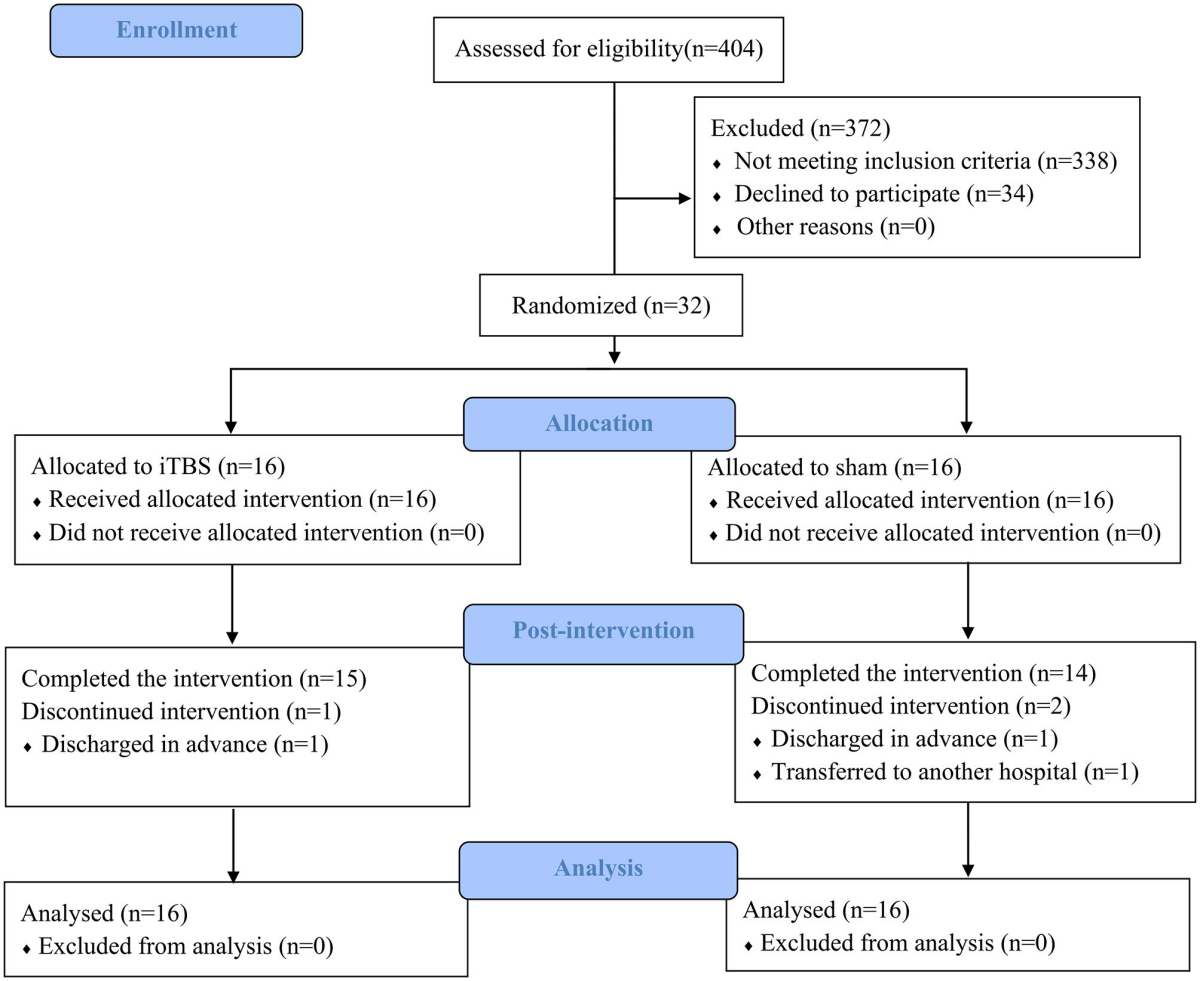
Consolidated standards of reporting trials flow diagram of the trial. iTBS, intermittent theta-burst stimulation.

After the screening based on the inclusion and exclusion criteria, a total of 32 out of 404 patients recruited were identified as being eligible for the trial. The whole procedure was well-tolerated, and no adverse events were reported in either group. During the intervention period, three subjects withdrew because two subjects were discharged in advance and one subject was transferred to another hospital that is near home (Figure 1). At baseline, no significant between-group differences were found in age, sex, time since stroke, types of stroke, paretic side, and the severity of stroke assessed by NIHSS (Table 1).

**Table 1:** Characteristics of the participants.

**Variable**	**Cerebellar iTBS (*n* = 16)**	**Sham stimulation (*n* = 16)**	***P-*value**
Age (y), mean ± SD	57.38 ± 8.04	51.44 ± 9.19	0.061^a^
Gender: male, *n* (%)	13 (81.25%)	12 (75.00%)	>0.999^b^
Time since the stroke (d), mean ± SD	80.13 ± 35.19	101.50 ± 54.15	0.196^a^
Type of stroke: ischemic, *n* (%)	10 (62.50%)	8 (50.00%)	0.722^b^
Paretic side: left, *n* (%)	12 (75.00%)	7 (43.75%)	0.149^b^
NIHSS score, *n* (%)			0.685^b^
0~4	13 (81.25%)	11 (68.75%)	
5~15	3 (18.75%)	5 (31.25%)	

*iTBS, intermittent theta-burst stimulation; y, years; d, days; SD, standard deviation; NIHSS, National Institute of Health Stroke Scale*.

a *Analyzed by unpaired t-test*.

b *Analyzed by Fisher's exact test*.

### Outcome Measures

**Figure 2:**
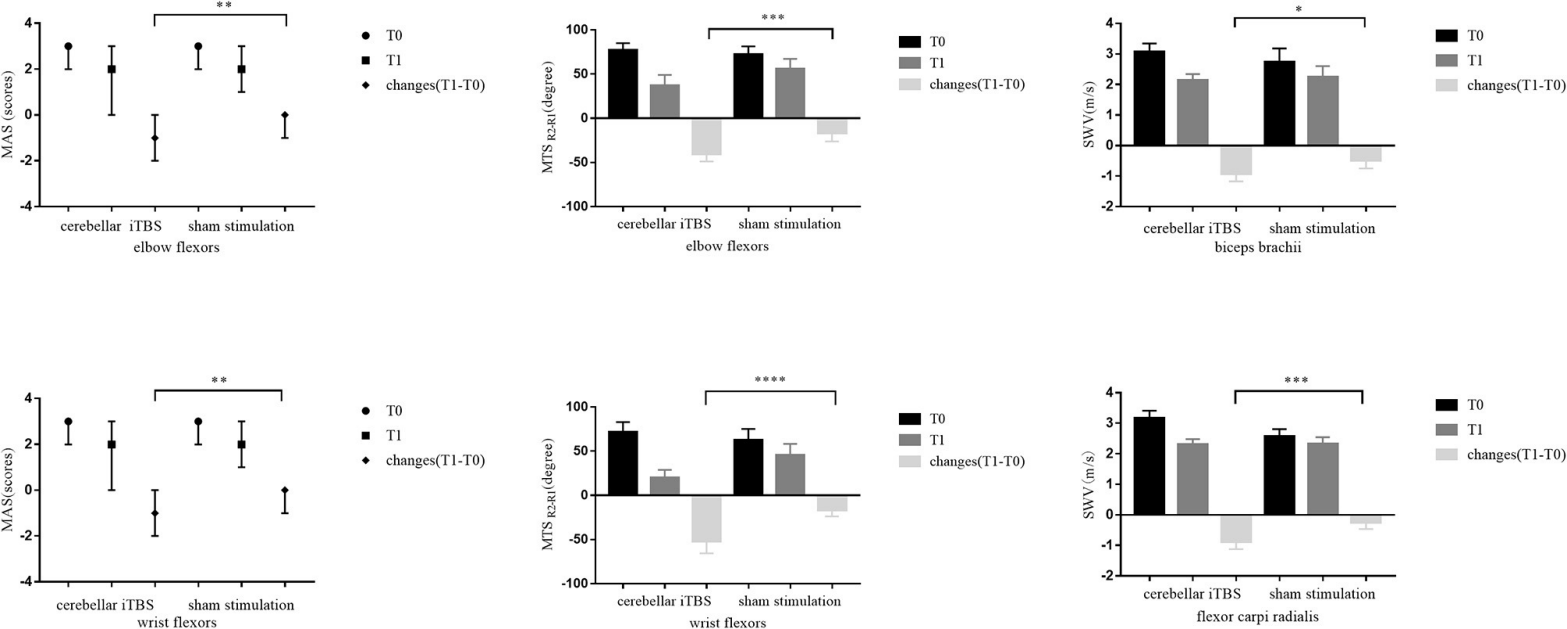
The statistical analysis results between groups for the primary outcomes. The data are expressed as the Median with IQR or Mean with 95%CI. *P < 0.05, **P < 0.01, ***P < 0.001, ****P < 0.0001.

Table 2 lists the descriptive data for all outcome measures in both groups at T0 and T1. Figure 2 provides the statistical analysis results between groups.

**Table 2:** The descriptive data for all outcome measures in both groups at T0 and T1.

**Outcome measures**	**T0**		**T1**		**Difference within groups (T1–T0)**	
	**Cerebellar iTBS**	**Sham stimulation**	**Cerebellar iTBS**	**Sham stimulation**	**Cerebellar iTBS**	**Sham stimulation**
MAS						
Elbow flexors	3.00 (3.00,3.00)	3.00 (2.00,3.00)	2.00 (2.00,2.00)	2.00 (2.00, 3.00)	−1.00 (−1.00, −1.00)^a^^,^ ^***^	0.00 (−1.00, 0.00)^a^^,^ ^*^
Wrist flexors	3.00 (2.75,3.00)	3.00 (2.00,3.00)	2.00 (1.00,2.00)	2.00 (2.00, 3.00)	−1.00 (−2.00, −1.00)^a^^,^ ^***^	0.00 (−1.00, 0.00)^a^
MTS (R2–R1) (deg.)						
Elbow flexors	80.00 (75.00, 88.00)	79.50 (57.50, 81.25)	36.73 ± 22.26	55.71 ± 19.91	−40.27 ± 15.29^a^^,^ ^***^	−16.36 ± 16.84^a^^,^ ^**^
Wrist flexors	71.57 ± 19.76	62.14 ± 22.45	19.64 ± 15.87	45.14 ± 22.64	−51.93 ± 23.55^b^^,^ ^***^	−17.00 ± 11.58^b^^,^ ^***^
SWV (m/s)						
Biceps brachii	3.07 ± 0.50	2.73 ± 0.75	2.15 ± 0.35	2.25 ± 0.59	−0.92 ± 0.45^b^^,^ ^***^	−0.48 ± 0.44^b^^,^ ^**^
Flexor carpi radialis	3.17 ± 0.42	2.57 ± 0.39	2.30 ± 0.32	2.32 ± 0.37	−0.87 ± 0.43^b^^,^ ^***^	−0.25 ± 0.35^b^^,^ ^*^
*H*_max_/*M*_max_ ratio	0.60 ± 0.49	0.79 ± 0.47	0.33 ± 0.26	0.46 ± 0.22	−0.05 (−0.53, 0.00)^b^	−0.33 ± 0.37^b^^,^ ^**^
MEP latency (ms)	22.14 ± 1.32	21.33 ± 2.21	21.72 ± 1.56	21.45 ± 2.10	−0.42 ± 1.24^b^	0.00 (−1.13, 0.73)^b^
MEP amplitude (μV)	140.47 ± 48.45	188.00 ± 97.41	170.00 (136.00, 238.67)	176.34 ± 69.01	30.67 (12.00, 62.00)^a^^,^ ^**^	2.67 (−43.97, 46.67)^b^
CMCT (ms)	8.17 ± 2.41	7.23 ± 2.36	6.37 ± 2.26	6.50 ± 2.34	−0.85 (−2.72, −0.17)^b^	−0.74 ± 2.91^b^
BI	60.00 ± 21.68	70.94 ± 13.32	69.06 ± 16.75	78.13 ± 12.76	9.06 ± 8.61^b^^,^ ^***^	7.19 ± 5.76^b^^,^ ^***^

*T0, at baseline; T1, after 10 sessions of intervention; iTBS, intermittent theta burst stimulation; MAS, modified Ashworth scale; MTS, modified Tardieu scale; deg., degree; SWV, shear wave velocity; H_max_/M_max_ ratio, H-maximum wave/M-maximum wave amplitude ratio; MEP, motor-evoked potential; CMCT, central motor conduction time; BI, the Barthel Index*.

* *Within group: P < 0.05, when compared with baseline*.

** *Within group: P < 0.01, when compared with baseline*.

*** *Within group: P < 0.001, when compared with baseline*.

a *Wilcoxon matched-pairs signed-rank test*.

b *Paired t-test*.

#### The Results of Primary Outcomes

The Mann–Whitney *U* test showed that 2 weeks of cerebellar iTBS coupled with conventional physical therapy resulted in the decreases of the MAS scores for affected elbow flexors and wrist flexors compared with sham stimulation (elbow flexors: *P* = 0.004, wrist flexors: *P* = 0.002). After the intervention, Wilcoxon matched-pairs signed-rank test revealed that both groups showed a significant decrease in the MAS scores of elbow flexors (cerebellar iTBS group: *P* < 0.001, sham stimulation group: *P* = 0.031), and the cerebellar iTBS group also showed the improvement in the MAS scores of wrist flexors (*P* < 0.001).

Consistent with the result of MAS, significant differences in MTS scores (elbow flexors: *P* < 0.001, wrist flexors: *P* < 0.0001) and SWV values (biceps brachii: *P* = 0.015, flexor carpi radialis: *P* < 0.001) of upper limb were also found between cerebellar iTBS and sham stimulation groups. The analysis of effectiveness within groups indicated that MTS scores and SWV values of upper limb significantly improved after interventions both in the cerebellar iTBS group (MTS scores: elbow flexors, *P* < 0.001; wrist flexors, *P* < 0.001. SWV values: biceps brachii, *P* < 0.001; flexor carpi radialis, *P* < 0.001) and the sham stimulation group (MTS scores: elbow flexors, *P* = *0*.005; wrist flexors, *P* < 0.001. SWV values: biceps brachii, *P* = 0.002; flexor carpi radialis, *P* = 0.023) (Figure 2).

#### The Results of Secondary Outcomes

**Figure 3:**
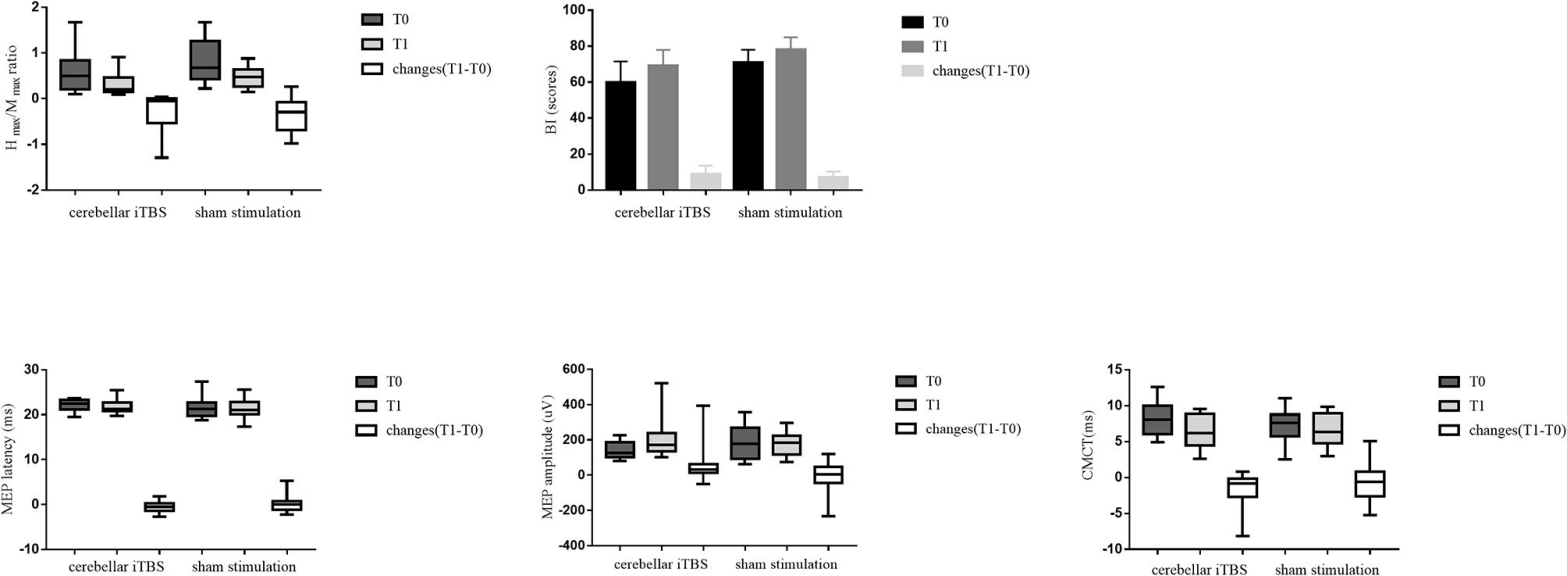
The statistical analysis results between groups for the secondary outcomes. The data are expressed as the Median with IQR or Mean with 95%CI.

After the intervention, the patients of both groups showed significant improvements in the BI (cerebellar iTBS group, *P* < 0.001; sham stimulation group, *P* < 0.001) scores compared with baseline. For the MEP amplitude, a significant increase was detected only in the cerebellar iTBS group at T1 compared with T0 (*P* = 0.003). For the *H*_max_/*M*_max_ ratio, a significant decrease was detected only in the sham stimulation group at T1 compared with T0 (*P* = 0.009). However, there were no differences between the groups for all the secondary outcomes (Figure 3).

## Discussion

This randomized, double-blind, sham-controlled study was designed to explore the short-term efficacy of cerebellar iTBS coupled with conventional physical therapy on upper limb spasticity in subacute stroke patients. Our results show that cerebellar iTBS coupled with conventional physical therapy improves PSS of the upper limb and ADL in individuals with stroke, as demonstrated by the decreased MAS and MTS scores, the reduced SWV values, and the increased BI scores after intervention. Importantly, the effectiveness of cerebellar iTBS on spasticity is promising, as significant improvements in the MAS scores, MTS scores, and SWV values were detected in the cerebellar iTBS group when compared with the sham stimulation group.

### The Effect of Cerebellar iTBS on Spasticity

Our study systematically assessed elbow flexors and wrist flexors spasticity by clinical, electrophysiological, and biomechanical measurements. Both clinical and biomechanical measurements showed significant improvements after cerebellar iTBS when compared with sham stimulation.

From a clinical point of view, we found that cerebellar iTBS coupled with conventional physical therapy decreased MAS scores of elbow flexors and wrist flexors from 3 to 2 points, passing from a marked increase level (2) to a slight increase level (1+) (Bohannon and Smith, 1987). In addition, the result of the difference between groups showed that the median changes of MAS scores of both elbow flexors and wrist flexors decreased one level in the cerebellar iTBS group when compared with the sham stimulation group, revealing that the efficacy of the cerebellar iTBS combined with conventional physical therapy was significantly better than that of the conventional physical therapy alone. Notably, the above changes reached the minimal clinically important difference that indicates a clinical significance was detected (Chen et al., 2019). Consistent with the result of MAS, MTS was also significantly improved by the 2 weeks of cerebellar iTBS coupled with conventional physical therapy, with the average angle differences between R2 and R1 of elbow flexors and wrist flexors reduced 43.00 degrees and 51.93 degrees, respectively. The range of motion at the upper limb is associated with cosmesis, hygiene, and active movement capabilities (Malhotra et al., 2011). Therefore, the increase in the range of motion at the upper limb can remove some participation restrictions and improve the quality of life in individuals with stroke.

From an electrophysiological point of view, spasticity is associated with the over hyperexcitability of spinal alpha motor neurons. The H-reflex is commonly used to quantify the level of spinal alpha motor neuron excitability (Pizzi et al., 2005). The *H*_max_/*M*_max_ ratio is proposed as a measure of the percentage of motoneurons activated by eliciting the monosynaptic H-reflex compared with those directly activated (Okuyama et al., 2018), which presents high reliability and good sensitivity in detecting changes in spasticity (Levin and Hui-Chan, 1993). In this study, no significant improvements in the *H*_max_/*M*_max_ ratio were found between groups. It may be attributed to the high variability of H-reflex in measuring the median nerve (Kim et al., 2015). Another possible reason is that the stimulation intensity of iTBS (80% aMT) was lower than the intensity of conventional rTMS (100% rMT) to induce electrophysiological changes in PSS improvement. Kim et al. also reported similar findings that iTBS over the affected motor cortex did not affect the H-reflex evoked in flexor carpi radialis (Kim et al., 2015).

From a biomechanical point of view, shear wave ultrasound elastography is feasible in muscle hardness assessment that may offer a better quantification of spasticity compared with clinical and electrophysiological measurements (Gao et al., 2018; Chen et al., 2019). In contrast to normal muscle cells, spastic muscle cells from stroke had shorter resting sarcomeres and increased elastic moduli, indicating that muscle stiffness can reflect the disease-related alterations in tissue properties (Wu et al., 2017). As shear waves travel faster in stiffer tissues, greater SWV and echo intensity were detected in spastic muscles (Lee et al., 2015). Our data demonstrated that the average SWV values of both biceps brachii and flexor carpi radialis were decreased in both groups after the intervention, and the cerebellar iTBS coupled with conventional physical therapy significantly decreased more SWV than sham stimulation. It implied that the therapeutic effect of cerebellar iTBS on changing muscle tissue properties was relatively obvious.

In agreement with relevant reported research published in 2014 (Barros Galvao et al., 2014), it was found that physiotherapy combined with additional low-frequency rTMS on unaffected primary motor cortex was more effective than physiotherapy alone in reducing upper limb spasticity in patients with chronic stroke (Barros Galvao et al., 2014). Furthermore, the benefits of iTBS on the affected motor cortex as an effective intervention to improve PSS had been identified by Kim et al. (2015). Their research found that a single session of iTBS contributed to a transient improvement in upper limb spasticity after stroke, which proved that iTBS seems to be an effective adjuvant to manage upper limb spasticity. Consistent with the above-reported studies, our study revealed significant improvements in upper limb spasticity after cerebellar iTBS in individuals with subacute stroke.

It is important to point out that the mean age of the subjects in the cerebellar iTBS group was 5.94 years older than that in the sham stimulation group, although it did not reach statistical significance. Age is a significant predictor of upper limb spasticity after stroke, with an odds ratio of 0.01 (Tedesco Triccas et al., 2019). The increase in aging individuals could have an impact on a higher incidence of upper limb spasticity. Besides, age is also a critical factor for predicting stroke outcome, with a negative correlation between age and the score of the motor component of the Functional Independence Measure and an active correlation between age and the length of hospital stay (Koyama et al., 2020). In our study, a significant decrease in upper limb spasticity was detected in the older cerebellar iTBS group patients.

### Possible Mechanism

The mechanism of iTBS over the ipsilesional lateral cerebellum reducing upper limb spasticity is still unclear. iTBS consists of high-frequency stimulation bursts, and the stimulus pattern is based on the natural theta rhythm occurring in the hippocampus of the brain, which can strongly modulate the neural activity of the cerebellum (Klomjai et al., 2015; Koch et al., 2019). An animal study reported that there were two modes of synaptic plasticity in the cerebellum, long-term depression (LTD) and long-term potentiation (LTP) (Jörntell and Hansel, 2006). Cerebellar iTBS can induce plasticity changes in the cerebellum of stroke patients (Koch et al., 2018). Besides, the molecular evidence also suggested that high-frequency rTMS could induce neural plasticity in the cerebellum associated with LTD (Lee et al., 2014). iTBS over the ipsilesional lateral cerebellum can increase the activation of Purkinje cells; the inhibitory synaptic connections between Purkinje cells and deep cerebellar nuclei are enhanced so that the neural plasticity in the cerebellum has been regulated (Fernandez et al., 2018). Besides, cerebellum iTBS can influence the activities of spinal neurons involved in the muscle tone adjustments in two ways: the inhibitory synaptic connections between Purkinje cells and dentate nucleus reduce the tonic excitatory effect of the dentate nucleus over the contralateral cerebral cortex through the ventrolateral nucleus of the thalamus; alternatively, interposed nuclei (globose and emboliform) and fastigial nucleus directly affect both the medial and lateral descending motor systems to reduce muscle spasticity (Teixeira et al., 2015). The relevant descending pathways include the corticospinal, reticulospinal, vestibulospinal, rubrospinal, and tectospinal tracts (Matsugi and Okada, 2020). Considering the results of our study, no significant difference in corticospinal excitability assessments (MEP latency and amplitude, and CMCT) was observed between groups. Therefore, the cerebellar iTBS improved upper limb spasticity may attribute to promoting the functional connection between the cerebellum and other brain areas rather than concerting with the cerebral cortex.

Physiological evidence shows that the cerebellum can interfere in the muscle tone adjustment by regulating neuronal discharge in different brain stem nuclei, primarily the reticular formation, red nucleus, and vestibular nucleus. Besides, the vestibular nucleus is involved in the alpha motor neuron activation, while the reticular formation and red nucleus are involved in the gamma motor neuron activation (D'Angelo, 2018). Chothia et al. found that anodal cerebellar direct current stimulation regulated the reticular spinal tract and rubrospinal tract to affect the motor neurons of the spinal cord (Chothia et al., 2016).

Other than motor areas, cerebellar iTBS also affects non-motor areas. Casula et al. found that the induction of cerebellar plasticity by iTBS was also associated with relevant changes in the neural activity of the posterior parietal cortex (Casula et al., 2016). The posterior parietal cortex participates in the perception and processing of action-related information and encodes the more abstract aspects of sensorimotor control processes, which is involved in the upper limb rehabilitation in patients with PSS (Veverka et al., 2019).

### The Effects of Cerebellar iTBS on ADL

In our study, increases in BI scores were shown in both groups after interventions. These effects are likely due to the course of coupled 2 weeks of daily conventional physical therapy, independently from the cerebellar iTBS treatment. Conventional physical therapy has been confirmed as an effective way for the recovery of function after stroke (McDonnell and Stinear, 2017), whereas iTBS is an adjuvant tool to reinforce the therapeutic effect of conventional physical therapy.

### Limitations

We acknowledge that some limitations still existed in this study. First, the lack of follow-up did not allow us to explore the long-term efficacy of cerebellar iTBS. Second, the potential mechanisms of cerebellar iTBS need to explore further to confirm our hypothesis. Therefore, high-quality randomized controlled trials with larger sample sizes to investigate the long-term efficacy and potential mechanisms of cerebellar iTBS are recommended for future studies.

## Conclusion

Our study is the first study to provide novel evidence that combining cerebellar iTBS with conventional physical therapy is an effective strategy to promote upper limb PSS recovery in patients with subacute stroke. The result of the effectiveness of cerebellar iTBS in terms of the MAS, MTS, and SWV at the upper limb is significant. Therefore, cerebellar iTBS is a promising adjuvant tool to reinforce the therapeutic effect of conventional physical therapy in spasticity management for patients after subacute stroke.

## Data Availability Statement

The original contributions presented in the study are included in the article/Supplementary Material, further inquiries can be directed to the corresponding author/s.

## Ethics Statement

The studies involving human participants were reviewed and approved by Biomedical Ethics Committee of West China Hospital, Sichuan University. The patients/participants provided their written informed consent to participate in this study.

## Author Contributions

YC and QG: concept, idea, research design, and writing. YC, Q-CW, and M-ZZ: research implementation. Y-JX and L-YL: data collection. H-XT and Q-FG: data analysis. QG: project management.

## Conflict of Interest

The authors declare that the research was conducted in the absence of any commercial or financial relationships that could be construed as a potential conflict of interest.

## Publisher's Note

All claims expressed in this article are solely those of the authors and do not necessarily represent those of their affiliated organizations, or those of the publisher, the editors and the reviewers. Any product that may be evaluated in this article, or claim that may be made by its manufacturer, is not guaranteed or endorsed by the publisher.
