# Supplementary material for: Cerebellar Intermittent Theta-Burst Stimulation Reduces Upper Limb Spasticity After Subacute Stroke: A Randomized Controlled Trial
Source: Front Neural Circuits. 2021 Oct 27;15:655502. doi: 10.3389/fncir.2021.655502 (PMC8578104; doi:10.3389/fncir.2021.655502)
Supplement: Supplementary file 1 [file Table_1.DOCX]

Supplementary Material

# Supplementary Table: conventional physical therapy program.

| **Training** | **Treatment Intervention** | **Time** |
| --- | --- | --- |
| limb positioning | The subject is in the supine position, with head on a pillow at an appropriate height which is slightly higher than the thoracic vertebrae. The therapist put his/her affected limb in a good position: scapular band extending forward, shoulder abduction, elbow extension, forearm supination, wrist and fingers extension, bilateral pelvis supinated slightly, the lower limb extension with the knee bent slightly. | 5 min |
| sensory stimulation | 1. Proprioceptive facilitation of the affected upper limb: the therapist stimulates the affected upper limb through compression, traction, rotation, movement, and contact to enhance proprioceptive input and promote the reaching and grasping activities when the patient is in the supine position. During this training, the range of motion is increased gradually. The subject is asked to maintain the limb in a certain position to improve the perception of the upper limb.  2. Plantar intrinsic muscles activation of the affected lower limb: the subject is in the supine position. The therapist uses both hands to contact the plantar intrinsic muscles and stimulates proprioceptors to activate the internal muscles. This training can improve the posture of the foot which contributes to standing balance. | 10 min |
| stretching | 1. Stretching training for the affected upper limb: the subject is in the supine position. The therapist stretches the subject's elbow and wrist flexors slowly and maintains for 30 seconds at the end of the range of motion.  2. Stretching training for the affected lower limb: the subject is in the supine position. The therapist stretches the subject's knee flexors and Achilles tendon slowly and maintains for 30 seconds at the end of the range of motion. | 10 min |
| task-oriented therapy | 1. Task-oriented training for upper limb: including unilateral (affected side) and bilateral functional exercises, activity of daily living goal, context specific environment using real-life object manipulation, and exercise in multiple movement planes.  2. Task-oriented balance training: including stepping up and down, heel-raising exercises, semi-squatting, and walking across obstacles. | 10 min |
| postural training | The therapist conducts postural training when the subject is in various positions based on the Bobath concept: including trunk control training, maintaining an unsupported sitting posture, sit-to-stand training, and gait training. | 15 min |

All subjects underwent a conventional physical therapy program immediately after cerebellar or sham iTBS, lasting 50 minutes per session. This program was composed of exercises designed to improve spasticity and promote recovery of voluntary motor function of the upper limb. Therapy is flexible, responsive, and patient-centered such that its starting point and progression are influenced by factors such as functional level, environmental context, and the individual’s perceived needs.

**
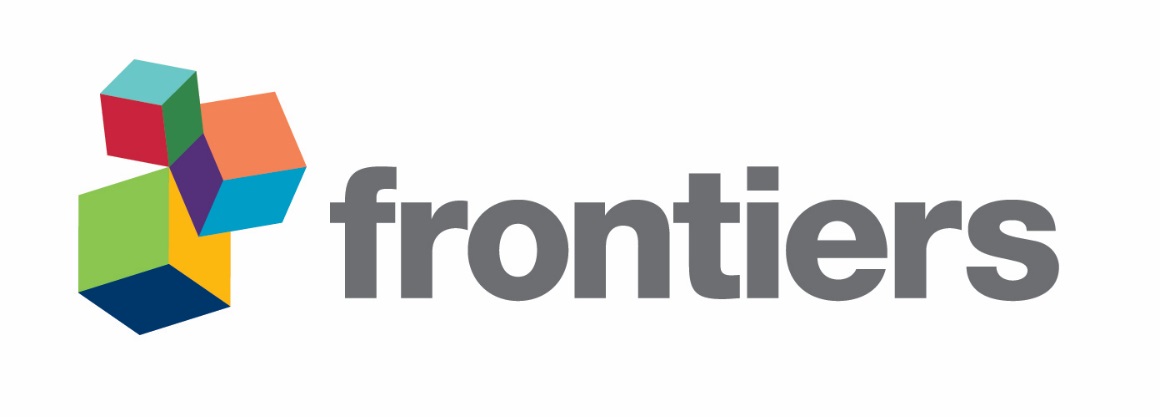
**
